# Imaging and clinical data archive for head and neck squamous cell carcinoma patients treated with radiotherapy

**DOI:** 10.1038/sdata.2018.173

**Published:** 2018-09-04

**Authors:** Aaron J. Grossberg, Abdallah S. R. Mohamed, Hesham El Halawani, William C. Bennett, Kirk E. Smith, Tracy S. Nolan, Bowman Williams, Sasikarn Chamchod, Jolien Heukelom, Michael E. Kantor, Theodora Browne, Katherine A. Hutcheson, G. Brandon Gunn, Adam S. Garden, William H. Morrison, Steven J. Frank, David I. Rosenthal, John B. Freymann, Clifton D. Fuller

**Affiliations:** 1 Department of Radiation Oncology, The University of Texas MD Anderson Cancer Center, Houston, Texas 77030, USA; 2 Department of Radiation Medicine, Oregon Health and Science University, Portland, Oregon 97238, USA; 3 Department of Clinical Oncology and Nuclear Medicine, Faculty of Medicine, University of Alexandria, Alexandria, 21321 Egypt; 4 Department of Biomedical Informatics, University of Arkansas for Medical Sciences, Little Rock, Arkansas 72205, USA; 5 Radiation Oncology Unit, Chulabhorn Hospital, Bangkok 10210, Thailand; 6 Department of Radiation Oncology, Netherlands Cancer Institute, 1066 CX Amsterdam, The Netherlands; 7 Department of Head and Neck Surgery, The University of Texas MD Anderson Cancer Center, Houston, Texas 77030, USA; 8 Leidos Biomedical Research, Inc. Frederick National Laboratory for Cancer Research, Frederick, Maryland 20892, USA

**Keywords:** Head and neck cancer, Cancer imaging, Radiotherapy

## Abstract

Cross sectional imaging is essential for the patient-specific planning and delivery of radiotherapy, a primary determinant of head and neck cancer outcomes. Due to challenges ensuring data quality and patient de-identification, publicly available datasets including diagnostic and radiation treatment planning imaging are scarce. In this data descriptor, we detail the collection and processing of computed tomography based imaging in 215 patients with head and neck squamous cell carcinoma that were treated with radiotherapy. Using cross sectional imaging, we calculated total body skeletal muscle and adipose content before and after treatment. We detail techniques for validating the high quality of these data and describe the processes of data de-identification and transfer. All imaging data are subject- and date-matched to clinical data from each patient, including demographics, risk factors, grade, stage, recurrence, and survival. These data are a valuable resource for studying the association between patient-specific anatomic and metabolic features, treatment planning, and oncologic outcomes, and the first that allows for the integration of body composition as a risk factor or study outcome.

## Background & Summary

Head and neck cancer accounts for more than 350,000 cases annually worldwide^1^, and about 3–4% of malignancies in the United States and Europe, accounting for over 200,000 cancer deaths per year^2,3^. Outcomes for patients with head and neck squamous cell carcinoma (HNSCC) have changed markedly over the past two decades, reflecting evolving patterns of risk factors (tobacco, human papillomavirus [HPV], and Epstein-Barr virus) and technical evolutions in radiotherapy (RT)^4–6^. However, the staging system for this set of diseases still reflects a primarily surgical approach, providing limited modern day prognostic value and leading to efforts to define more predictive outcome modelling. Such analyses have shown factors such as age, smoking history, human papillomavirus status, and, more recently, skeletal muscle depletion provide greater predictive value than American Joint Committee on Cancer staging alone^4,7^.

Furthermore, advances in cancer imaging, RT planning and delivery allow for increasingly conformal and individualized treatments. However, these treatment details are poorly reflected in reports from large multicentre randomized trials, in which the impact of RT is addressed as a binary factor. The absence of treatment specifics, including disease delineation, treatment volume identification, and dose delivery, limit the identification of patient and treatment factors critical to both controlling disease and limiting toxicity^8^. Publicly shared RT data are scarce due to high complexity of RT structure data and the need for registration in time, space, and across planning sets. Recent advances in data archiving, patient de-identification, and image registration have allowed for the creation of a high quality RT-enriched imaging database within The Cancer Imaging Archive (TCIA)^9,10^.

**Figure 1:**
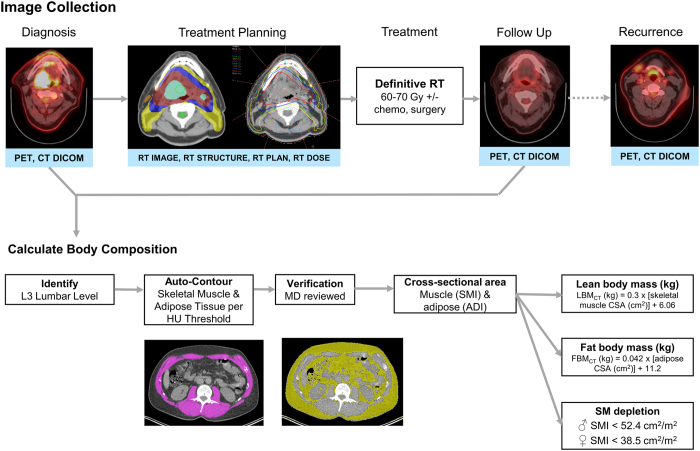
Schema representation of image collection and analysis workflow.

Here we present the curation of the HNSCC collection, a single-institutional collection of 215 patients treated definitively for HNSCC at The University of Texas MD Anderson Cancer Center from 2003 to 2013. The HNSCC Collection integrates deidentified, comprehensive clinical data with diagnostic imaging, RT structure, planning, and dose deposition data, and makes it publicly available to researchers. Unique to this dataset is the inclusion of CT-derived body composition estimates, both before and after treatment, allowing for the inclusion of body composition as either a risk factor or endpoint. Subject selection and data collection are summarized in Fig. 1. These data were used in a prior publication, which evaluated the association between body composition and oncologic outcomes in head and neck cancer patients treated with radiotherapy^7^. The open access to these data allows for interinstitutional comparisons of non-randomized patient, treatment, and outcome data that would not otherwise be possible.

## Methods

### Patient selection and clinical end points

To develop this dataset, the records of 2840 consecutive patients with HNSCC treated with curative-intent RT at The University of Texas MD Anderson Cancer Center from October 1, 2003, to August 31, 2013, were screened. Those patients with whole-body PET-CT or abdominal CT scans performed within the parent institution both before and after RT were included (n=215). This study was approved by the institutional review board of the University of Texas MD Anderson Cancer Center. Written informed consent was obtained from all study participants. Clinical data were stored in and retrieved from the MD Anderson Cancer Center custom electronic medical record system, ClinicStation. The majority of the patients were male (85.5%). One hundred and fifty-six of 215 patients (73%) had tumors arising in the oropharynx, with the most common sites being the base of the tongue (51%) and tonsil (43%). One hundred twenty seven patients (59%) received concurrent chemotherapy, with 98% of these patients receiving platinum-based systemic treatment. Twenty-seven patients (13%) received radiation in the postoperative setting. Mean radiation dose was 68.66 Gy (range, 56–72 Gy) delivered in 28–40 daily fractions. Detailed clinical characteristics of the patients are saved on the TCIA website in tabular format (Patient and Treatment Characteristics, Data Citation 1). Description of the patient and clinical characteristics table are stored on the TCIA website (Field Descriptions for Patient and Treatment Characteristics, Data Citation 1).

Tumor histology, grade, and HPV association was assessed by pathology at the parent institution. HPV status was assessed using *in situ* hybridization for high risk HPV subtypes. For patients diagnosed at external medical centers, central pathology review was provided. Staging for all cancers was assigned according to the 7^th^ edition of the AJCC Staging Manual using the TNM system. Overall survival was defined as the time from diagnosis to the date of death due to any cause. Disease-specific survival was defined as the time from diagnosis to death due to HNSCC; deaths due to other causes were censored. Duration of locoregional control was defined as the time from diagnosis to the date of locoregional recurrence. Distant recurrences and second primary tumors were censored. Smoking history was coded as “never smoker” (0), “less than 10 pack-years” (1), or “greater than or equal to 10 pack-years” (2), and current smoking status was coded as “no” (0) or “yes” (1). Feeding tube provision, including both nasogastric and percutaneous placement, at any time during or following RT was recorded dichotomously, with type of feeding tube, dates of placement and removal, and feeding tube duration noted.

### Image Acquisition

All images in this dataset were acquired at MD Anderson Cancer Center. Initial diagnostic imaging was obtained either at the time of diagnosis or the time of presentation to MD Anderson, if diagnosis occurred at an outside institution. Whole-body PET-CT scans were used for the majority of patients (n=212, 98.6%), as part of standard staging workup to identify tumor metastases to regional lymph nodes or distant organs. Patients with distant metastases at the time of diagnosis (Stage IVC) were excluded from the data set. Non-contrast radiation therapy simulation CT scans were obtained immediately prior to the start of RT for treatment planning purposes. The median time between initial diagnostic imaging and simulation was 0.87 months (interquartile range 0.37–2.27 months), dependent upon whether the patient received neoadjuvant chemotherapy or primary surgery prior to RT. Follow up whole-body PET-CT (n=213, 99.1%) or abdominal CT scans (n=2) were collected per treating physician discretion, with a median interval between RT completion and follow up imaging of 2.77 months (interquartile range 1.93–6.57 months). Follow up imaging was most commonly obtained to evaluate treatment response approximately 2–3 months after RT completion, however, in some cases was ordered to evaluate tumor recurrence or metastasis, leading to longer intervals between treatment and imaging. Recurrence imaging was collected by CT, PET/CT or MRI. PET/CT scans were obtained on GE Medical Systems Discovery RX, Discovery ST, and Discovery STE hybrid PET/CT scanners after the intravenous injection of ^18^F-labeled fluorodeoxyglucose (FDG). CT scans including the abdominal region were obtained using GE Medical Systems LightSpeed or Discovery CT750HD scanners. Radiation treatment planning simulation scans were acquired using Picker PQ 5000, Marconi MX8000, and Philips Brilliance Big Bore CT scanners-- those that were in use at MD Anderson during the period of data capture. MRI scans were obtained using GE Medical Systems Genesis Signa, Signa Excite, and Signa HDxt scanners. Complete imaging details for each DICOM, including image type, date, study description, scanner and software details, and CT protocol and reconstruction details are stored on the TCIA website in tabular format (DICOM Imaging Output Details, Data Citation 1). The CT images follow the standard DICOM format are organized by anonymized patient ID number (TCIA code), and can be cross-referenced against the data table using this identifier. Detailed description of each data field, including DICOM tag information, is listed in Table 1. Because DICOM-RT files are generated in treatment planning software and not obtained from an imaging device, scanner manufacturer for each of these files is listed as “Pinnacle”.

Diagnostic images were stored in the MD Anderson PACS system as DICOM files. Images were exported, as DICOM, directly from the PACS server into a research server for collation prior to file transfer to TCIA. CT simulation, RTSTRUCT, RTDOSE, and RTPLAN files were stored in the institution’s clinical Pinnacle server until treatment is completed. After 12 weeks without modifications, DICOM files were archived, verified, and then deleted from Pinnacle. During archival, secondary image sets’ DICOM data were removed because it is duplicated from other systems and also contained within the archive in a different format. Archived files were then retrieved and extracted into a research institution that interacts with Pinnacle from a different server. RTDOSE and RTPLAN are then recalculated and all files are exported as DICOM files prior to transmission to TCIA. Therefore, RT planning DICOM files in this data set are generated from Pinnacle during the export process.

### Radiation Treatment Planning

Immediately following simulation CT acquisition, images were pushed into the Pinnacle image server for target delineation and treatment planning (version 9, Philips Radiation Oncology Systems, Fitchburg, WI). Contours were drawn on axial images to delineate target volumes in accordance with International Commission on Radiation Units and Measurements (ICRU) Reports 50^11^ and 62^12^. All contours were then reviewed in the head and neck radiation oncology section quality assurance conference, during which patients returned to clinic for physical examination by multiple physicians with fiberoptic nasopharyngolaryngoscopy, as needed, followed by critical review of target volumes. After modification, contours were submitted to dosimetry for treatment planning. Radiation technique was dependent on prescribing physician preference, and included conventional 2D radiation therapy, intensity modulated radiation therapy (IMRT) matched to 2D in the mid-neck using half-beam block technique, whole-field IMRT, and volumetric arc therapy. Isodose lines and dose volume histograms were then reviewed by the prescribing physician, with any changes in treatment planning implemented and reviewed. Treatment plans then underwent a quality assurance step performed by medical physicists to ensure feasibility of dose delivery and accurate dose deposition using phantoms before treatment commencement.

### Body Composition Analysis

The methodology we utilized to determine body composition is described in our previous manuscript and recounted here^7^. Briefly, we analysed pre- and post-treatment CT images of the third lumbar vertebra (L3), including abdominal CT scans and the CT component of whole-body PET-CT scans^7,13,14^. Images were analysed using the radiation treatment planning system, Pinnacle version 9.6 (Philips Radiation Oncology Systems, Fitchburg, WI). Three adjacent axial images within the same series were selected for analysis of total muscle cross-sectional area (in cm^2^), and the mean cross-sectional area was calculated for each patient by dividing the contoured volume by the slice thickness. Skeletal muscle was defined by a Hounsfield unit range of −29 to 150^15^, and adipose tissue by a range of −190 to −30 Hounsfield units^16,17^. These thresholds were used for autosegmentation, after which the contours were manually reviewed and corrected by a single radiation oncologist with 5 years of post-residency clinical experience. Contoured skeletal muscles included the rectus abdominus, abdominal wall, psoas, and paraspinal muscle groups. Iso-attenuating intra-abdominal organs were edited out of the contoured volumes. Adipose tissue cross sectional area included intra-abdominal and subcutaneous, but not intramuscular adipose tissue. The cross-sectional area of muscle and adipose tissue was then normalized to the square of height in meters, as with body mass index (BMI), and reported as the lumbar skeletal muscle index (SMI) or adipose index (ADI) in units of cm^2^/m^2^ ^7,14,18,19^. Skeletal muscle depletion was defined as an SMI<52.4 cm^2^/m^2^ for men and<38.5 cm^2^/m^2^ for women, a threshold that is associated with poor prognosis in cancer patients^7,18,19^. Each patient was coded as having “depleted” or “not depleted” skeletal muscle at both the pre- and post-treatment imaging time points. Total body lean body mass, comprising bone and soft tissue, and fat mass were estimated from L3 skeletal muscle and adipose tissue cross sectional areas from pre- and post-treatment scans using the following formulae^14^:

$$\mathrm{LBM}\left(\mathrm{kg}\right)=0.3\times \left[\mathrm{skeletal}\;\mathrm{muscle}\;\mathrm{cross}\;\mathrm{sectional}\;\mathrm{area}\;\mathrm{at}\;\mathrm{L3}\left({\mathrm{cm}}^{2}\right)\right]+6.06$$

$$\mathrm{FM}\left(\mathrm{kg}\right)=0.042\times \left[\mathrm{total}\;\mathrm{adipose}\;\mathrm{tissue}\;\mathrm{cross}\;\mathrm{sectional}\;\mathrm{area}\;\mathrm{at}\;\mathrm{L3}\left({\mathrm{cm}}^{2}\right)\right]+11.2$$

### Data transmission and de-identification

Data transmission to TCIA and patient de-identification were conducted using the Radiologic Society of North America (RSNA) Medical Imaging Resource Center (MIRC) Clinical Trial Processor (CTP) program. CTP is a stand-alone program that combines DICOM export from the primary institution, DICOM anonymization, and subsequent DICOM import and file storage. Detailed description of the program and its components can be found at http://mircwiki.rsna.org/index.php?title=MIRC_CTP (2016). Following data transmission CTP modifies the DICOM to delete personal health information (PHI) in accordance with the Health Insurance Portability and Accountability Act (HIPAA), as defined by the DICOM standards committee Attribute Confidentiality Profile (DICOM PS 3.15: Appendix E, ftp://medical.nema.org/medical/dicom/final/sup142_ft.pdf), which describes a standard procedure and documentation for removal of PHI from DICOM images^20^. A complete description of the steps used in PHI removal is found at the TCIA de-identification knowledge base website, https://wiki.cancerimagingarchive.net/display/Public/De-identification+Knowledge+Base (2014). CTP de-identification proceeded according to the following workflow:

1. CTP was installed at both the data acquisition site (MD Anderson Cancer Center) and the server site (TCIA).
2. Unique patient identifier numbers are assigned to each subject.
3. Initial de-identification according to the Basic Attribute Confidentiality Profile. This included the following considerations:
4. Removal of PHI information from descriptive tags containing unstructured plain text values over which an operator has control.
5. Modification of tags that contain dates or times with a standard date offset applied to all images, allowing retention of inter-scan and inter-subject temporal interval information.
6. Hashing of UIDs.
7. Retention of newly assigned patient identifiers.
8. Retention of physical characteristics of the patient that are descriptive but not personally identifying information, such as metabolic measures, body weight, etc.
9. Retention of information about the characteristics of the device used to perform the image acquisition.
10. Private Attributes confirmed not to contain PHI are retained.
11. Visual inspection of each image in the collection by the collection curator to guarantee that no PHI is burned in to the pixel data
12. Tag Sniffer, a tool that scans DICOM images for unique strings in the image header, annotated a list of DICOM elements for review after comparing elements to the Basic Application Confidentiality Profile.
13. Elements not designated for removal per the Basic Application Confidentiality Profile were then reviewed by the TCIA collection curator and all PHI elements, non-hashed UIDs, and non-offset dates were identified. This list was then reviewed by the collection manager, who made final decisions regarding the removal or retention of these elements.
14. A custom CTP script was then written to anonymize additional PHI elements identified above.
15. After application of the CTP script, Tag Sniffer was used again to review the images and create the Final Review Output. This list was reviewed by the collection curator and manager to ensure that no PHI was carried forward after de-identification.

For curation of this dataset, CTP was installed and utilized without modification, with the exception of the final transfer, during which the burned-in PHI filter was disabled for transfer. Burned in PHI elements were then identified during the above listed quality control process and subsequently removed.

### Code availability

Body composition analyses were performed using the commercially available radiation treatment planning software, Pinnacle, version 9.6 (Philips Radiation Oncology Systems, Fitchburg, WI).

The code underlying the CTP DICOM file transfer and de-identification process is openly available from the RSNA MIRC website: http://mirc.rsna.org/query. To download the software, follow the link in the left panel and save the CTP-installer.jar file.

The code for the Tag Sniffer DICOM de-identification software is openly available: https://code.imphub.org/projects/TCIA/repos/dicomtagsniffer/browse.

The Posda Tools code, responsible for technical validation and generation of descriptive tables for the DICOM collection, is openly available, and contributions from the research community are encouraged: https://github.com/UAMS-DBMI/PosdaTools.

## Data Records

The HNSCC collection is a dataset consisting of 433,384 DICOM files from 3,225 series and 765 studies collected from 215 patients, as well as a single XLSX file including all of the demographic, clinical, treatment, and body-composition data for each patient (Data Citation 1). Images are organized by anonymized patient ID number (SUBJECT_ID) and can be cross-referenced against the data table using this identifier. For each patient there is a pre-treatment diagnostic CT or PET/CT study, a simulation CT study, RTPLAN (showing beams, collimation, energy, and monitor units delivered), RTSTRUCT (showing physician- and dosimetrist-contoured target and avoidance volumes), and RTDOSE (showing isodose lines), and a post-treatment diagnostic CT or PET/CT study. For those patients who had loco-regional recurrence, diagnostic CT or PET/CT studies used to diagnose their recurrence are also included.

## Technical Validation

One of the major challenges in curating an imaging database including DICOM images generated from multiple manufacturers and processed across multiple clinical and research pipelines, and subsequent de-identification, is the loss of important information that may impact reliability and interpretation. In this data set the most common errors involved inconsistencies between series and studies, and improperly linked data sets, causing misregistration between RT structure sets and source DICOM imaging. To ensure data have not been altered, technical validation was performed using Posda Tools (https://github.com/UAMS-DBMI/PosdaTools; 2016), a web-based open-access software written in perl developed for curation of images for TCIA^21^.

Posda Tools are a large application based on Posda DICOM toolkit, which includes a DICOM receiver feature, modules for parsing, dumping, and modifying DICOM files, and a file indexing function that can import improperly tagged files into the database, such that study and series consistency can be queried. Because Posda has not been thoroughly described in prior literature, we introduce some of its features below.

### Architecture of Posda Tools

The architecture of the Posda Tools is based on a number of different features. These are primarily the following:

1. The “tag signature” model for referencing DICOM tags embedded within nested sequences.
2. The Posda DICOM toolkit
3. The Posda DICOM/file database
4. File Digest based directory hierarchies
5. An event driven programming environment based on the “select” system call and “no wait I/O”.
6. The Posda web-based application framework based on State-full processes with sessions and named objects within sessions addressable via a RESTful API.
7. The Posda “Nicknames” module for creating persistent short names within the context of a submission, site, and subject which can be used in place of UIDs for Study, Series, Instance, and Frame of Reference. This idea was inherited from the DICOMpiler tool^22,23^.
8. The Posda DICOM Application Entities.
9. Resource locking via transaction manager.
10. Application Launcher as a web-based application.
11. Parallelism based upon forking of UNIX sub-processes.
12. Storing of complex state in serialized perl data structures.
13. Tracking of all changes by revision with full audit trail.
14. Support for “Incidental” DICOM edit scripts within a tracked, audit trail environment.

The Posda toolkit, at present, is a very large collection of perl include files and programs which use these files. As a rough measure of its size, there are 165 perl include files with a total line count of 62,817 lines (this excludes a DICOM Data Dictionary object which consists of 74,018 lines). It also contains 328 perl programs with a total line count of 38,569 lines. The Posda Curation App consists of 11 perl include file with a total line count of 8,230. It contains 34 perl programs with a total line count of 4,934.

### Using Posda Tools

Posda Tools received images from CTP and presented them to the curator based on the collection name, site name, and subject name (Patient ID). DICOM files were then screened for errors and edited to correct any issues that are detected. The typical workflow for this process was as follows:

1. Extract and view errors. Errors were generated when extracted files have inappropriate element values or an incorrect number of values for a given element.
2. Determine whether multiple files are duplicates. Duplicate files were identified by selecting two files and using the “compare” function. Differing only in UIDs and creation dates is highly suggestive of duplicate files.
3. Delete duplicates.
4. Move CTs related to RT Structure Set into RT Study.
5. Move incorrect CTs out of RT Study.
6. Relink RTSTRUCT/RTDOSE/RTPLAN to CT files
7. Eliminate inconsistencies. Inconsistencies are fixed using the bulk “Fix Study Inconsistencies” function, which proposed a fix for all study inconsistencies that impacts the fewest number of files. These were then manually reviewed and performed as appropriate.
8. Repeat Steps 1–7 until no errors are found.
9. Confirm that structure sets are appropriately linked by importing files into a structure viewer (also based on Posda).
10. Edit Frame of Reference UID in the relinked structure sets to match the Frame of Reference UID in the CT files and Structure Set.
11. Extract and export descriptive data from each DICOM file into a spreadsheet.
12. In the data presented in this paper, initial analysis showed that 195 of the 215 subjects had series and study inconsistency problems. At completion of curation, the minimum number of revisions for a subject was 2, the maximum was 6, and the average number of revisions was 3.17.

## Usage Notes

The HNSCC Collection is provided as a collection of DICOM files with accompanying (CSV) files. Use of this data set is open to all researchers in accordance with TCIA usage policies. When citing this TCIA collection, both the DOI for this collection and this data descriptor should be cited.

Images are stored in standard DICOM format and may be viewed and analysed in any DICOM viewing application, depending on the end user’s requirements. For example, 3D Slicer (http://slicer.org) is a free and open source platform for medical image visualization and quantitative analysis. The TCIA Browser extension of 3D Slicer enables integration of the versatile visualization and computing tools of 3D Slicer with unique data resources of TCIA.

## Additional information

**How to cite this article**: Grossberg, A. J. *et al*., Imaging and clinical data archive for head and neck squamous cell carcinoma patients treated with radiotherapy. *Sci. Data* 5:180173 doi: 10.1038/sdata.2018.173 (2018).

**Publisher’s note**: Springer Nature remains neutral with regard to jurisdictional claims in published maps and institutional affiliations.

## Supplementary Material



## Figures and Tables

**Table 1:** Description of data categories in (DICOM Imaging Output Detail, Data Citation 1).

Data Category	Description (Tag)
SUBJECT_ID	Patient ID number randomly assigned to each patient prior to anonymizing the DICOM PHI tag (0010,0020)
**ImageType**	Origin of retrieved DICOM image file. ORIGINAL=DICOM file directly collected from scanner; DERIVED=reconstructed or reformatted DICOM file; NULL=DICOM-RT file constructed from RT planning software (0008,0008)
**Modality**	Type of equipment that originally acquired the data used to create the images in this Series. CT=computed tomography; PT=positron emission tomography; MR=magnetic resonance imaging; RTPLAN=beams, collimation, energy, and monitor units delivered; RTSTRUCT=physician- and dosimetrist-contoured target and avoidance volumes, RTDOSE=isodose lines (0008,0060)
**Images**	Number of images in the study (0020,1002)
**StudyDate**	Date study was performed after standard date offset was applied to original DICOM file. (0008,0020)
**StudyDescription**	Institution-generated description or classification of the Study (component) performed. (0008,1030)
**SeriesDescription**	Description of series from each study. (0008,103E)
**SeriesNumber**	Numerical assignment for each series. (0020,0011)
**StudyInstanceUID**	Unique ID number for study generated during DICOM anonymization (0020,000D)
**SeriesInstanceUID**	Unique ID number for series generated during DICOM anonymization (0020,000E)
**Mfr**	Manufacturer of scanner used to obtain image. DICOM-RT files are generated in treatment planning software and listed as ADAC. (0008,0070)
**Model**	Model of scanner used to obtain DICOM files. DICOM-RT files are generated in treatment planning software and listed as Pinnacle3. (0008,1090)
**software_versions**	Software version used for construction of the DICOM file. (0018,1020)
**DicomObjectType**	Type of DICOM object (0008,0008)
**kvp**	X-ray tube voltage used for the acquisition of DICOM file (0018,0060)
**scan_options**	Parameters of scanning sequence. (0018,0022)
**data_collection_diameter**	The diameter in mm of the region over which data were collected (0018,0022)
**reconstruction_diameter**	Diameter in mm of the region from within which data were used in creating the reconstruction of the image. Data may exist outside this region and portions of the patient may exist outside this region. (0018,1100)
**dist_source_to_detect**	Distance in mm from source to detector center (0018,1110)
**gantry_tilt**	Angle of tilt in degrees of the scanning gantry. (degrees). (0018,1120)
**rotation_dir**	Direction of rotation of the source about nearest principal axis of equipment. CW=clockwise CC=counter clockwise (0018,1140)
**exposure_time**	Time of x-ray exposure in msec (0018,1150)
**table_feed_per_rot**	Motion of the table (in mm) during a complete revolution of the source around the gantry orbit. (0018,9310)
**radiopharmaceutical**	Radiopharmaceutical used in positron-emission tomography scan. (0018,0031)
**total_dose**	The radiopharmaceutical dose administered to the patient measured in MegaBecquerels (MBq) (0018,1074).
**half_life**	Half life of radiopharmaceutical, in seconds.(0018,1075)
**positron_fraction**	The radionuclide positron fraction (fraction of decays that are by positron emission)(0018,1076)
**fov_shape**	Field of view shape. (0018,1147)
**fov_dim**	Field of view diameter specified for image acquisition. (0018,1149)
**coll_type**	Collimator type. (0018,1181)
**recon_diam**	Field of view diameter specified for PET image reconstruction (0018,1181)
