# Author Correction: Imaging and clinical data archive for head and neck squamous cell carcinoma patients treated with radiotherapy

**DOI:** 10.1038/s41597-018-0002-5

**Published:** 2018-11-27

**Authors:** Aaron J. Grossberg, Abdallah S. R. Mohamed, Hesham Elhalawani, William C. Bennett, Kirk E. Smith, Tracy S. Nolan, Bowman Williams, Sasikarn Chamchod, Jolien Heukelom, Michael E. Kantor, Theodora Browne, Katherine A. Hutcheson, G. Brandon Gunn, Adam S. Garden, William H. Morrison, Steven J. Frank, David I. Rosenthal, John B. Freymann, Clifton D. Fuller

**Affiliations:** 1 Department of Radiation Oncology, The University of Texas MD Anderson Cancer Center, Houston, Texas 77030 USA; 2 Department of Radiation Medicine, Oregon Health and Science University, Portland, Oregon 97238 USA; 3 Department of Clinical Oncology and Nuclear Medicine, Faculty of Medicine, University of Alexandria, Alexandria, 21321 Egypt; 4 Department of Biomedical Informatics, University of Arkansas for Medical Sciences, Little Rock, Arkansas 72205 USA; 5 Radiation Oncology Unit, Chulabhorn Hospital, Bangkok, 10210 Thailand; 6 Department of Radiation Oncology, Netherlands Cancer Institute, 1066 CX Amsterdam, The Netherlands; 7 Department of Head and Neck Surgery, The University of Texas MD Anderson Cancer Center, Houston, Texas 77030 USA; 8 Leidos Biomedical Research, Inc., Frederick National Laboratory for Cancer Research, Frederick, Maryland 20892 USA

Correction to: *Scientific Data*, 10.1038/sdata.2018.173, Published online 4 September 2018

In the original version of the Data Descriptor the surname of author Hesham Elhalawani was misspelled. This has now been corrected in the HTML and PDF versions.

